# Modified procedure for reconstructing the inferomedial orbital wall: silicone sheet implantation without surgical removal

**DOI:** 10.1007/s00405-023-08257-6

**Published:** 2023-10-13

**Authors:** Kosuke Takabayashi, Yohei Maeda, Nobuya Kataoka

**Affiliations:** 1 Department of Otorhinolaryngology, Japanese Red Cross Asahikawa Hospital, Asahikawa City, Hokkaido Japan; 2 Department of Otorhinolaryngology, Sapporo Medical University School of Medicine, Sapporo City, Hokkaido Japan; 3 Department of Otorhinolaryngology, Japan Community Health Care Organization Osaka Hospital, 4-2-78 Fukushima, Fukushima-Ku, Osaka City, Osaka 553-0003 Japan; 4 Department of Otorhinolaryngology–Head and Neck Surgery, Osaka University Graduate School of Medicine, Suita City, Osaka Japan; 5 Department of Ophthalmology, Japanese Red Cross Asahikawa Hospital, Asahikawa City, Hokkaido Japan

**Keywords:** Silicone sheet, Balloon, Bone-mucosal flap, Transorbital approach, Transnasal approach

## Abstract

**Background:**

Due to the complexity of reconstructing wide inferomedial orbital wall fractures, silicone sheets are the preferred choice of reconstructive material. Nevertheless, it is crucial to remove the silicone sheet postoperatively due to the risk of delayed complications associated with its placement.

**Methods:**

We developed a procedure in which a silicone sheet implanted in the orbit can be extracted through the nasal cavity by removing the fractured portion of the medial orbital wall.

**Conclusion:**

This procedure enables the utilization of silicone sheets, which are suitable for intricate orbital reconstruction, without any concerns regarding delayed complications.

**Supplementary Information:**

The online version of this article (10.1007/s00405-023-08257-6) contains supplementary material, which is available to authorized users.

## Relevant surgical anatomy

Reconstruction of the inferomedial wall of the orbit after orbital blowout fracture is complicated due to the wide area of the fracture and the elaborate three-dimensional anatomy. Silicone sheets are a suitable material for complex orbital reconstruction due to their soft and pliable nature, which allows for easy creation of three-dimensional structures and reduces the risk of complications such as infections or extrusions [1].

Typically, the removal of the silicone sheet implanted in the orbit necessitates a second surgery. However, in this procedure, access to the silicone sheet is possible through the nasal cavity by removing the fractured portion on the medial wall of the orbit, eliminating the second surgery for silicone sheet removal.

## Description of the technique

**Fig. 1:**
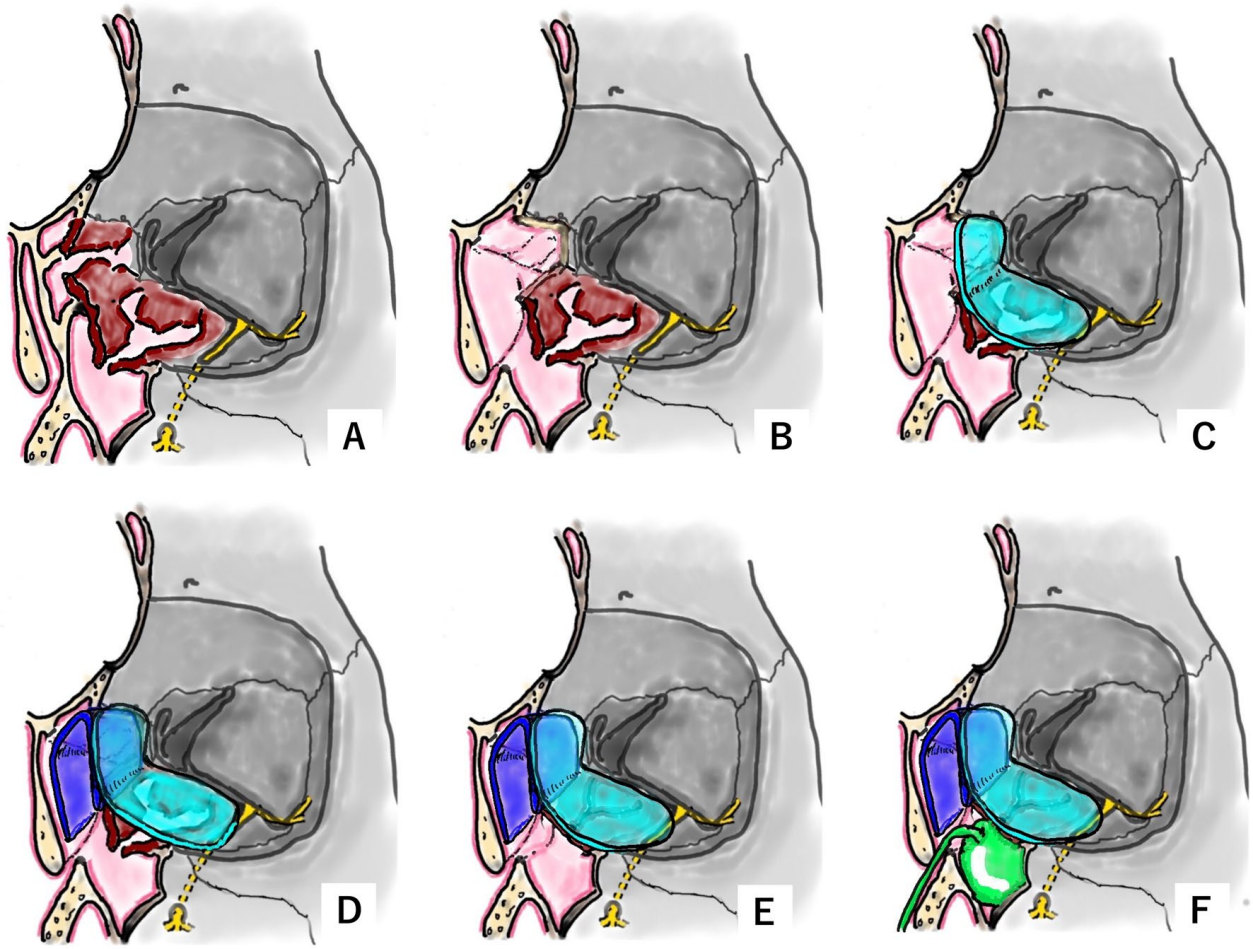
Schema showing steps of the double silicone procedure. **A** Inferomedial orbital wall fracture. Brown areas indicate the fractured inferomedial orbital wall. **B** Fractured bone fragments of the medial wall are removed, while those of the inferior wall are preserved as much as possible. **C** The transparent turquoise structure indicates the first silicone sheet implanted along the orbit. **D** The transparent blue structure indicates the second silicone sheet implanted in the ethmoid sinus. **E** The bone-mucosal flap of the infraorbital wall has been relocated to its original position. **F** The balloon is implanted in the maxillary sinus and the infraorbital wall is fixed. The green structure indicates the balloon

**Fig. 2:**
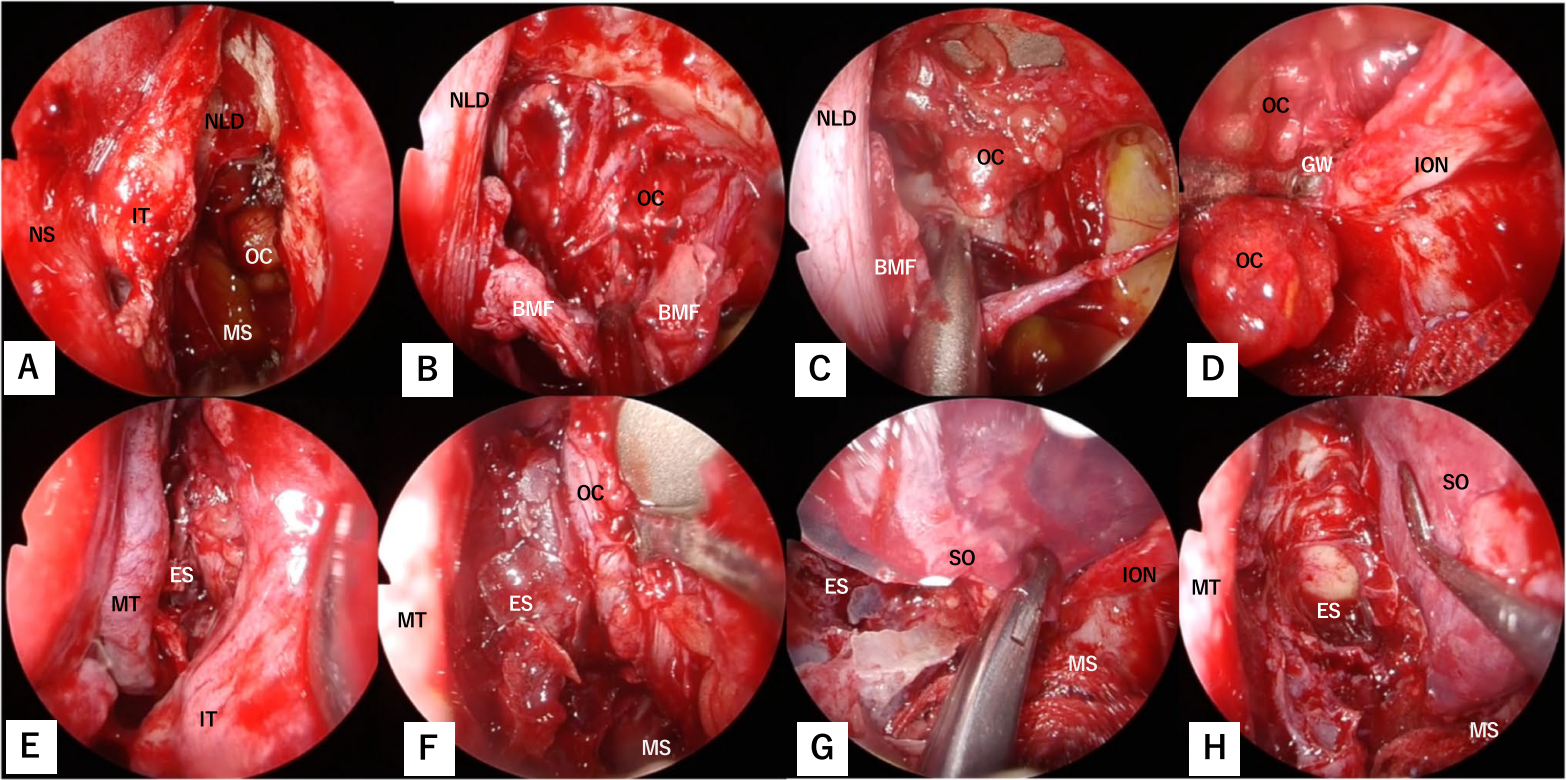
Endoscopic images of the procedure up to the placement of the first silicone sheet along the orbit. **A** The inside of the left maxillary sinus is observed from the prelacrimal approach. **B** The bone and mucosa at the fracture site of the infraorbital wall are elevated as a pedicled bone-mucosal flap to release adhesions with the orbital contents. **C** The transnasal surgeon and transorbital surgeon collaborate to restore the orbital contents. **D** The correct orbital height is confirmed by identifying the infraorbital nerve and the inferior margin of the greater wing of the sphenoid bone. **E** The inferior turbinate is placed in its original position, and manipulation of the ethmoid sinus is initiated. **F** The ethmoid sinus has been opened and the bone and mucosa at the fracture site of the medial orbital wall are removed. The orbital contents are retracted laterally by the transorbital surgeon. **G** The transorbital surgeon inserts a 1-mm-thick silicone sheet into the orbit transorbitally; the endoscopist provides support and places the sheet in the proper position. **H** The silicone sheet is placed along the orbit. *BMF* bone-mucosal flap; *ES* ethmoid sinus; *GW* greater wing of sphenoid bone; *ION* infraorbital nerve; *IT* inferior turbinate; *MS* maxillary sinus; *MT* middle turbinate; *NLD* nasolacrimal duct; *NS* nasal septum; *OC* orbital contents; *SO* silicone sheet in the orbit

**Fig. 3:**
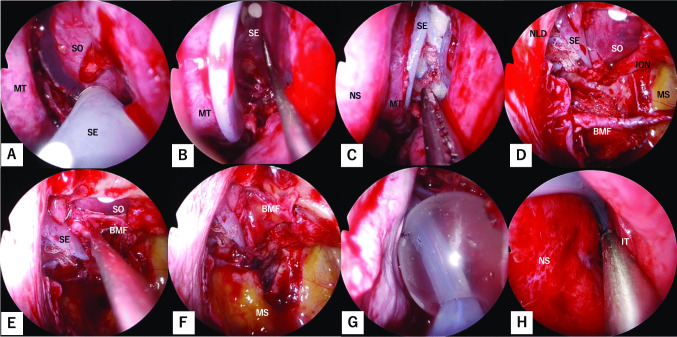
Stepwise endoscopic images of the procedure from the placement of the second silicone sheet to the end of surgery. **A** The second silicone sheet is placed into the ethmoid sinus by the transnasal surgeon. **B** The silicone sheet is implanted in the ethmoid sinus to form an inverted U-shape. **C** The inverted U-shaped silicone sheet is fixed in place by insertion of gauze coated with ointment. **D** After the silicone sheet is fixed in the ethmoid sinus, the maxillary sinus is manipulated. **E** The bone-mucosal flap is moved back to its normal position. **F** The orbit and maxillary sinus are completely separated by the bone-mucosal flap. **G** A balloon is placed in the maxillary sinus and the infraorbital wall is fixed. **H** The incision used in the prelacrimal approach is sutured, and the nasal cavity is restored. *BMF* bone-mucosal flap; *ION* infraorbital nerve; *IT* inferior turbinate; *MS* maxillary sinus; *MT* middle turbinate; *NLD* nasolacrimal duct; *NS* nasal septum; *SE* silicone sheet in the ethmoid sinus; *SO* silicone sheet in the orbit

Figure 1 shows the bony structures surrounding the left orbit and left nasal cavity with removal of the lacrimal bone, part of the maxilla, and part of the frontal bone. The inferomedial orbital wall fracture (Fig. 1A) was addressed using the combination of transorbital subciliary incision and an endoscopic transnasal approach to release adhesions and incarcerations. The first step was to manipulate the inferior wall, starting with a prelacrimal approach via the nasal cavity (Fig. 2A). The surgeons elevated the bone and maxillary sinus mucosa of the infraorbital wall at the fracture site as a pedicled local bone-mucosal flap (Fig. 2B, C), while the transorbital surgeon collaborated with them to restore the orbital contents (Fig. 2C). The height of the posterior margin of the orbital floor was accurately determined by identifying the infraorbital nerve, then proceeding posteriorly to locate the inferior margin of the greater wing of the sphenoid bone [2] (Fig. 2D). After completing the manipulation of the infraorbital wall, the ethmoid sinus was opened and the fractured bone fragments and mucosa of the medial orbital wall were removed [3, 4] (Fig. 1B). The orbital contents were retracted laterally by the transorbital surgeon, allowing the transnasal surgeon to safely manipulate the medial orbital wall (Fig. 2E, F). The transorbital surgeon inserted a 1-mm-thick silicone sheet (Eyeball Restraint Insert; Koken, Tokyo, Japan) into the orbit, positioning it along the inferior wall to the medial side, while the transnasal surgeon was responsible for reconstructing the medial wall, especially the deep medial part of the orbit and the side closest to the skull base (Figs. 1C, 2G, H). While the transorbital surgeon held the silicone sheet in place along the orbit, the transnasal surgeon placed another 1-mm-thick silicone sheet in an inverted U-shape in the opened ethmoid sinus; gauze coated with ointment was applied to secure the sheet along the orbit and prevent it from deviating medially (Figs. 1D, 3A–C). Once the silicone sheet was securely positioned, the transnasal surgeon restored the pedicled local bone-mucosal flap of the infraorbital wall to its original position (Figs. 1E, 3D–F). The inferior wall of the maxillary sinus was stabilized by inflating a balloon and confirming that it was in the correct position (Figs. 1F, 3G). The mucosal incision made during the prelacrimal approach was then closed with sutures (Fig. 3H).

**Fig. 4:**
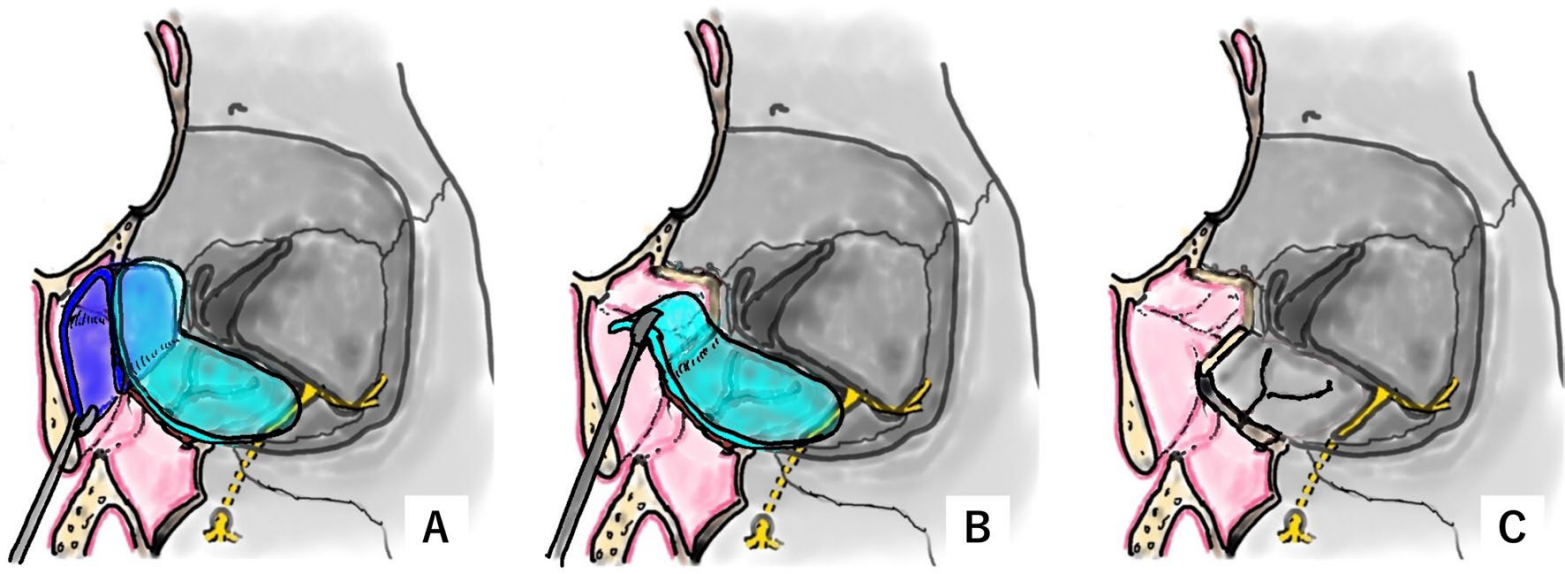
Schema showing steps of silicone sheet removal. **A** The second silicone sheet (transparent turquoise) implanted in the ethmoid sinus is grasped with forceps. **B** The first silicone sheet (transparent blue) implanted in the orbit is being pulled out into the ethmoid sinus with forceps. **C** The structure of the reconstructed orbit. The fractured area of the medial wall has been removed

**Fig. 5:**
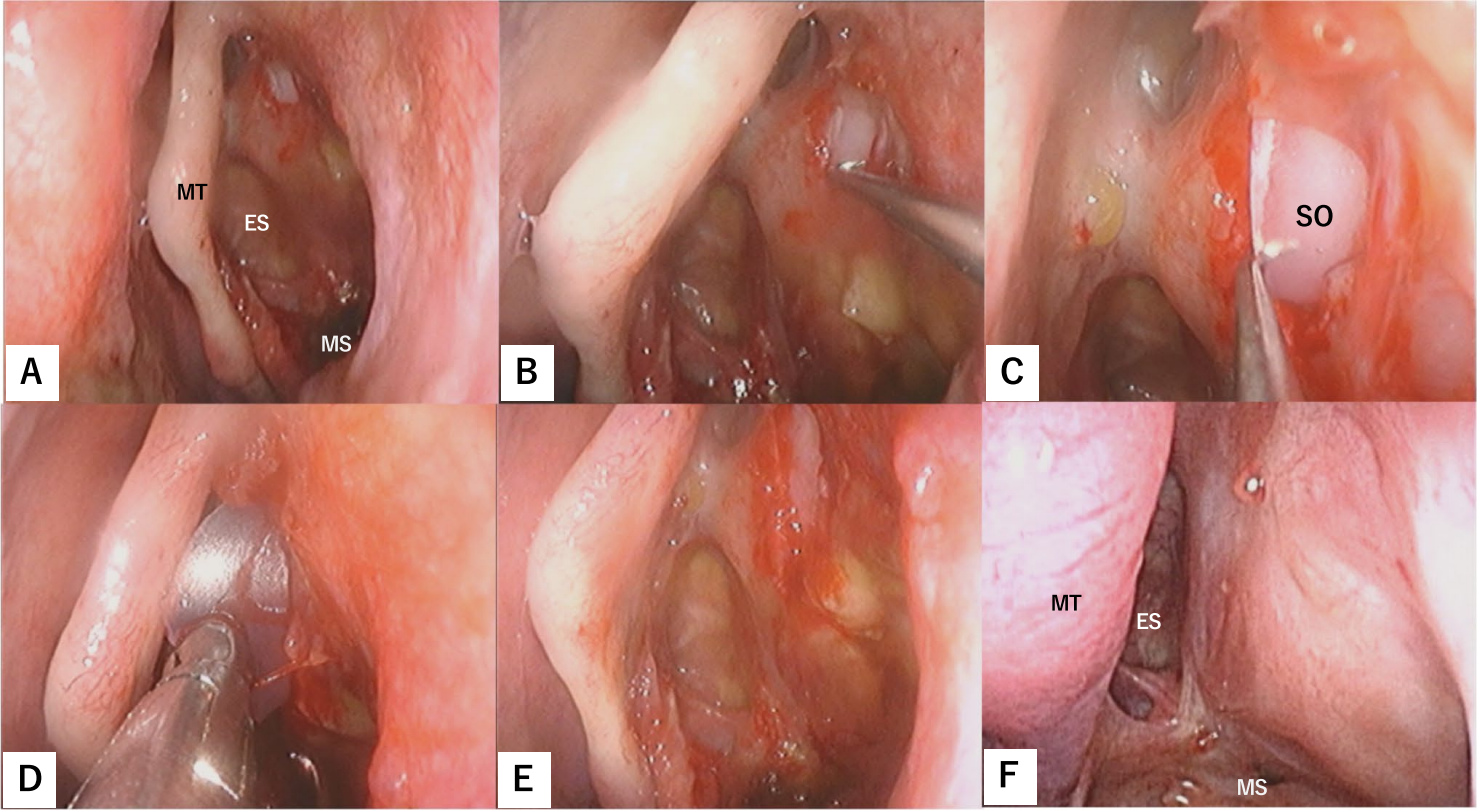
Endoscopic images of removal of the silicone sheet implanted along the orbit. **A** Endoscopic findings after removal of a silicone sheet that had been implanted in the ethmoid sinus. The silicone sheet along the orbit is partially visible on the medial orbit wall. **B**, **C** The silicone sheet is hooked and exposed in the ethmoid sinus. **D** The silicone sheet exposed in the ethmoid sinus is pulled out with forceps. **E** No deviation of the orbital contents occurs after removal of the silicone sheet. **F** Six months postoperatively, epithelialization of the ethmoid sinus and medial orbital wall is complete, and the ethmoid sinus space has been secured. *ES* ethmoid sinus; *MS* maxillary sinus; *MT* middle turbinate; *SO* silicone sheet in the orbit

The patient was discharged 1 week postoperatively when the balloon and the gauze with ointment were removed (Fig. 1E). One month postoperatively, the silicone sheets that were implanted intranasally and intraorbitally were removed in an outpatient procedure using topical anesthesia. First, the silicone sheet implanted in the ethmoid sinus was removed transnasally with forceps (Fig. 4A). The silicone sheet that was placed intraorbitally could be identified and removed transnasally using hooks or forceps (Figs. 4B, C, 5A–E). The silicone sheet can be readily extracted when the anteroposterior diameter of the fracture site in the medial orbital wall measures at least 2 cm. The silicone sheet is easily deformed and can be extracted even when the diameter is less than the specified dimension. A fibrous membrane had formed around the silicone sheet [5], with no deviation of the orbital contents. Six months postoperatively, epithelialization of the ethmoid sinus and medial orbital wall was complete, and the ethmoid sinus space was secured (Fig. 5F).

**Fig. 6:**
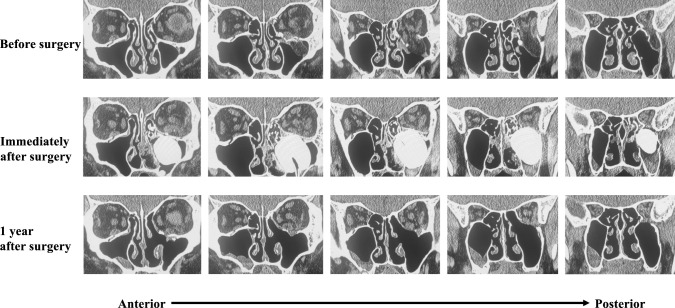
Comparison of computed tomography (CT) images before surgery, immediately afterward, and 1 year afterward. The upper, middle, and lower sections show CT images before surgery, immediately afterward, and 1 year afterward, respectively. The most anterior slices are shown on the left, and the most posterior slices are shown on the right. The orbit is well repaired 1 year after surgery

The orbital contents, which had deviated medially and inferiorly before surgery, were restored within the orbit, and the orbit was reconstructed with two silicone sheets and a balloon. One year postoperatively, the orbit was successfully reconstructed (Fig. 6).

## Discussion

Our newly developed technique named the “double silicone procedure” represents a breakthrough that overcomes the disadvantage of requiring a second surgery to remove silicone sheets used in inferomedial orbital wall reconstruction. Silicone sheets are an attractive option for orbital reconstruction due to their flexibility and ability to easily conform to a three-dimensional shape [1]. In our procedure, these sheets are removed through the nasal cavity on an outpatient basis, enabling orbital reconstruction without the need to consider delayed complications associated with their use, such as abscess formation, misalignment, and diplopia [5]. We would like to emphasize that this procedure is a safe and versatile technique for orbital reconstruction, and importantly it does not require rigid materials that are difficult to form and it is performed collaboratively by transnasal and transorbital surgeons.

This procedure achieves a similar orbit shape as surgery in which the fractured bone fragments are removed and the medial orbital wall is fixed with a silicone sheet [3, 4]. Silicone repair and fixation of the medial wall via intranasal manipulation have shown good results in previous studies, although there are potential complications such as enophthalmos, overcorrection, implant infection, and entrapment of orbital contents between the silicone sheet and the medial orbital wall [3, 4]. Based on the outcomes in the aforementioned previous studies, the final orbit shape achieved by this procedure is acceptable. Additionally, removing bone fragments from the medial wall eliminates the need for a second surgery to remove the silicone sheets used in reconstruction, which is a significant advantage of this procedure.

The fact that combined transorbital and endoscopic transnasal approaches are performed by two surgeons enhances the simplicity, safety, and accuracy of the operation since the surgeons can support each other. If the tissue deviation of the inferior wall is severe, the transorbital and transnasal surgeons should collaborate in the reconstruction using a prelacrimal approach to the maxillary sinus, whereas if the fracture is minor, the transorbital surgeon can perform the reconstruction of the inferior wall alone, thereby simplifying the procedure. In the medial wall manipulation, the transorbital surgeon provides lateral traction of the orbital contents, which greatly improves maneuverability for the endoscopist operating using one hand.

Recent developments have led to the emergence of alloplastic and resorbable rigid reconstructive materials that eliminate the need for a second surgery for removal and have demonstrated efficacy in orbital reconstruction [6]. However, complex inferomedial wall fractures necessitate three-dimensional reconstruction, making it challenging to shape a fractured orbit using rigid reconstructive materials. We wish to underscore that our technique, which employs silicone sheets, enables a broader range of surgeons to perform safe and successful three-dimensional orbital reconstruction.

## Indications

This procedure is indicated for patients with wide inferomedial orbital wall fractures.

## Limitations

Removal of the fractured portion of the medial orbital wall may lead to enophthalmos, particularly when the medial wall is extensively fractured.

## How to avoid complications

To prevent intrusion into the intraconal space, it is essential to identify and reconstruct the following landmarks: the infraorbital nerve, the inferior margin of the greater wing of the sphenoid bone, and the superior posterior wall of the maxillary sinus.

## Specific perioperative considerations

It is important to take note of optic neuropathy and intraorbital hematoma.

## Specific information to give to the patient about surgery and potential risks

Silicone sheets are the preferred material for reconstructing extensive and complex orbital wall fractures; however, their removal is necessary to prevent delayed complications. By contrast, this procedure eliminates the need for a second surgery to remove the silicone sheets.

## A summary of ten key points

1. This procedure employs silicone sheets that are deemed safe for reconstructing intricate orbital shapes.
2. While it is preferable to remove the silicone sheet to prevent late complications, this procedure eliminates the need for a second surgery specifically for silicone sheet removal.
3. By removing the fractured bone in the medial orbital wall, access to the silicone sheet implanted in the orbit can be achieved through the nasal cavity.
4. In cases of severe infraorbital wall fractures, reconstruction of the infraorbital wall involves collaboration between a transorbital surgeon and a transnasal surgeon.
5. For minor infraorbital wall fractures, the transorbital surgeon can independently perform the reconstruction of the infraorbital wall, streamlining the surgical procedure.
6. To ensure the safe reconstruction of the inferior wall, it is crucial to identify the following landmarks: the infraorbital nerve, the inferior margin of the greater wing of the sphenoid bone, and the superior posterior wall of the maxillary sinus.
7. During medial orbital maneuvers, the transorbital surgeon can apply outward traction on the deviated orbital contents, thereby enhancing the maneuverability of the transorbital surgeon.
8. The initial silicone piece is positioned along the orbit, extending from the inferior wall to the medial side.
9. The second silicone sheet is inserted in an inverted U-shape into the ethmoid sinus and securely fixed to prevent any displacement of the first silicone sheet from the orbit.
10. The silicone sheet can be removed through an outpatient procedure. Once the first silicone sheet is extracted, the second silicone sheet becomes visible on the orbital side and can be subsequently pulled out.

### Supplementary Information

Below is the link to the electronic supplementary material.

Supplementary file1 (MP4 111110 KB)

## Data Availability

The data that support the findings of this study are available from the corresponding author upon reasonable request.
